# Comprehensive dataset for hybrid mesoscopic modeling of adsorption rate dynamics and surface–fluid interactions

**DOI:** 10.1016/j.dib.2025.112403

**Published:** 2025-12-22

**Authors:** David DYCZKO, Damien Ali Hamada FAKRA, Rijalalaina RAKOTOSAONA, Marie Hanitriniaina RATSIMBA, Mino Patricia RANDRIANARISON

**Affiliations:** a Université de Lorraine, INRAE, Laboratory for Studies and Research on Wood Materials (LERMaB), F-54000 Nancy, France; b University of Antananarivo, Higher Polytechnic School of Antananarivo (ESPA), Research Laboratory of Material, Process and Civil Engineering (LRMPGC), Sis Ambohitsaina BP 1500, Antananarivo 101, Madagascar

**Keywords:** Adsorber, Sorption cycle, Hybrid model validation, optimal parameters determination

## Abstract

A new hybrid mesoscopic RC model has been developed to describe dynamic adsorption cycles on isothermal surfaces. The model has been rigorously validated through three independent campaigns: one inter-model comparison and two validations based on published scientific studies. This article consolidates the reference datasets employed in these validation exercises and provides the computational codes necessary to extract and calibrate the parameters of the proposed model. The results confirm the robustness and general applicability of the hybrid approach, establishing a reliable framework for the simulation and optimization of adsorption/desorption processes across diverse adsorbent systems.

Specifications Table

**Table:** 

Subject	Engineering & Materials science
Specific subject area	Data used for the validation of a new hybrid model of adsorption cycles that determines the quantity of adsorbate and the temperature of the adsorber
Type of data	Table, Chart, Graph, Code Raw, Discretized
Data collection	For sorption-mass-temperature.py, Fette [1] originally developed a numerical code capable of simulating both the adsorbate uptake *X* and the adsorber surface temperature T using the Dubinin–Astakhov isotherm combined with a Linear Driving Force (LDF) kinetic model. In the present work, this code was extended to incorporate all the comparative models evaluated in the study. The resulting simulated datasets are provided in models.csv. To obtain reference data for validation, graphical outputs were extracted from Figures 4 and 5 in Amin [2] and from Figure 7 in Brancato et al. [3]. The original images were first enhanced (contrast and saturation adjustments) using Polarr Pro in order to improve curve readability. Numerical data were then digitized automatically using WebPlotDigitizer (automeris.io), version 5. Finally, all extracted series were time-aligned and discretized in Microsoft Excel to ensure consistency with the selected simulation time step.
Data source location	Université de Lorraine, LERMaB, France
Data accessibility	Repository name: **Comprehensive dataset for hybrid mesoscopic modeling of adsorption rate dynamics and surface–fluid interactions** Data identification number: 10.17632/49zng74g9f.2 Direct URL to data: https://data.mendeley.com/datasets/49zng74g9f/2 *.py files written with Spyder 6 *.xlsx and *.csv files written with Excel Version 16.89.1
Related research article	D. Dyczko, D. Fakra, R. Djedjig, M. Khelifa, Hybrid Mesoscopic RC Modeling of Dynamic Adsorption Cycle on Isothermal Surfaces (manuscript submitted on Nov 23, 2025 in International Journal of Heat and Mass Transfer, Elsevier with the reference number: HMT-d-25–06,895) Preprint: https://papers.ssrn.com/sol3/papers.cfm?abstract_id=5812040

## 1 Value of the Data

• These datasets provide the essential foundation for validating a new hybrid dynamic model capable of simultaneously estimating the adsorbate mass fraction X and the surface temperature T of any adsorber.
• They offer complementary information that enhances the understanding of the associated research article in International Journal of Heat and Mass Transfer (reference: **HMT-d-25–06,895**) and under review.
• The Python programs included in this repository give detailed insight into the numerical implementation, providing a higher level of transparency and reproducibility than the standalone algorithms described in the associated article.
• All codes are fully operational and allow parameter configuration, enabling direct application to any adsorber or operating conditions.
• The spreadsheets and scripts can be readily adapted and reused in other research studies involving adsorption/desorption cycles or sorber modeling.

## 2 Background

This document provides a detailed explanation of the procedures employed to calculate the datasets used in the validation of the newly developed hybrid model for adsorber cycles. The model, which has been submitted for publication, required rigorous data processing to ensure consistency and reliability. Accordingly, the present document consolidates the computational steps and methodological choices underlying the generation of the validation data, thereby offering transparency and reproducibility for future research.

## 3 Data Description

**Table:** 

File	Tab	Comment	Illustration
sorption-mass-temperature.py		Python code for estimating *X* and *T* across seven adsorber models	
models.csv		Dataset of raw values from the seven models	
comparison-models.py		Parameter optimization of the hybrid model and assessment of simulation uncertainty through comparison with existing reference models	
Amin.xlsx	graphics	Graphical representations generated using WebPlotDigitizer (Automeris)	
	raw from chart	Numerical dataset derived from figures appearing in published scientific paper of Amin et al.	
	calculations	Time step alignment of Data: 10 s	
	data to export	Export Extracted Data to Amin.csv	
Amin.csv		Description about values data: + Col A corresponding of X values in kg adsorbate/kg adsorbent; + And, Col B corresponding of T in Kelvin	
Amin.py		Parameter optimization of the hybrid model based on Amin.csv with error analysis	
Brancato.xlsx	graphics	Graphical representations generated using WebPlotDigitizer (Automeris)	
	raw from chart	Numerical dataset derived from figures appearing in published scientific paper of Brancato et al.	
	calculations	Time step alignment of Data: 60 s	
	data to export	Export Extracted Data to Brancato.csv	
Brancato.csv		Description about values data: + Col A corresponding of X values in kg adsorbate/kg adsorbent; + And, Col B corresponding of T in Kelvin	
Brancato.py		Parameter Optimization of the New Model Based on Brancato.csv with error analysis	

## 4 Experimental Design, Materials and Methods

Inspired by the Python program created by N. Fette available at this link: https://www.mycompiler.io/view/DTj7zH0xIB6, a new program has been written to simulate the amount of adsorbate X (in kg adsorbate/kg adsorbent) and the temperature T (K) of an adsorber over several complete cycles of adsorption-desorption. This code allows the simulation of these X and T outputs according to seven different dynamic models: Freundlich associated with an LDF dynamics (*F*+LDF), Dubinin-Radushkevich (DR+LDF), Dubinin-Astakhov (DA+LDF), Saha-Boelman-Kashiwagi (SBK+LDF), DA with a Logarithmic Mean Temperature Difference dynamics (DA+LMTD), DA with the Finite Volume Method (DA+FVM), and the new hybrid model. The whole of this program is in file **sorption-mass-temperature.py** and requires the CoolProp module (library of thermophysical properties of fluids) to work. At the end of the code execution, the graphs representing the quantity of adsorbate X and the temperature T are drawn. The table **models.csv** exports the data for each of the models as well as the average of the six reference models (*F*+LDF, DR+LDF, DA+LDF, SBK+LDF, DA+LMTD, DA+FVM). Please, note that in this case, the new model settings are determined manually. Then, the other programs (**comparison-models.py, Amin.py** and **Brancato.py**) are built on the same structure. It is to determine the optimal parameters of the new model in order to minimize the deviation from the reference data, respectively **models.csv, Amin.csv** and **Brancato.csv**. This optimization is achieved by the Broyden-Fletcher-Goldfarb-Shanno method with the Large-scale and Bound-constrained options (L-BFGS-B) and requires the scikit-learn module for least squares calculations. This method was chosen according to the article by Bemporad [4].

**Table:** 

Program	Inputs	Outputs

comparison-models.py/Amin.py/Brancato.py	**Durations**: t_ads, t_pH, t_des, t_pc, dt **Number of cycles** : num_cycles **Reference data for X and T**: models.csv/Amin.csv/Brancato.csv.	Optimization parameters of the hybrid model: k_c_, k_r_, k_s_, k_t_, c, X_min_, X_max_, T_cool_, T_exhaust_, C, R_a_, and R_d_ Predicted X and T from the Hybrid Dynamic Model Deviation metrics for *X* and *T*: MAE, MRE, RMSE, Pearson *r*

To obtain the reference data corresponding to the studies by Amin and Brancato et al., the web tool WebPlotDigitizer (see https://automeris.io) was used to digitize the Figures. The extracted data were then discretized to match a chosen time step (10 s for Amin et al., 60 s for Brancato et al.). Inflection points in the curves were used to determine the duration of each phase: adsorption, preheating, desorption, and precooling. For Amin’s study, the phase durations are identical for each cycle, whereas in the Brancato experiments, the durations vary between cycles. The complete data processing workflow, from the original graphs to the reference datasets, is documented in the Excel workbooks **Amin.xlsx** and **Brancato.xlsx**.

## Limitations

Not applicable

## Ethics Statement

The authors have read and follow the ethical requirements for publication in Data in Brief. They confirm that the current work does not involve human subjects, animal experiments, or any data collected from social media platforms.

## Credit Author Statement

**David DYCZKO**: Conceptualization, Data curation, Formal analysis, Investigation, Software, Validation, Writing – original draft; **Damien Ali Hamada FAKRA**: Conceptualization, Data curation, Formal analysis, Methodology, Project administration, Software, Supervision, Validation, Writing – review & editing; **Rijalalaina RAKOTOSAONA**: Validation, Writing – review & editing; **Marie Hanitriniaina RATSIMBA**: Visualization, Writing – review & editing; **Mino Patricia RANDRIANARISON**: Data curation, Writing – review & editing.

## Data Availability

Mendeley DataExcel (Original data). Mendeley Datadataset (Reference data).
